# The Evolution of Custom Subperiosteal Implants for Treatment of Partial or Complete Edentulism in Patients with Severe Alveolar Ridge Atrophy

**DOI:** 10.3390/jcm13123582

**Published:** 2024-06-19

**Authors:** Jan Łoginoff, Agata Majos, Marcin Elgalal

**Affiliations:** II Department of Radiology and Diagnostic Imaging, Central Teaching Hospital of the Medical University of Lodz, 92-213 Lodz, Poland; jan.loginoff@umed.lodz.pl (J.Ł.); agata.majos@umed.lodz.pl (A.M.)

**Keywords:** subperiosteal implants, custom-made implants, CAD-CAM technologies, computed tomography

## Abstract

Dental implants have always played an important role in dentistry and have been used to replace missing teeth since around 600 AD. They can be classified into three groups: endosteal, subperiosteal, and transosteal. Over time, different materials have been used to manufacture dental implants and these, in turn, can be divided into three groups: metals, ceramics, and polymers. Today, the most commonly used treatment for edentulism is the use of endosteal implants. However, such an approach cannot be used in patients with severe alveolar ridge atrophy and, in such cases, custom subperiosteal implants are an alternative. This review article focuses on historical developments and improvements that have been made over recent years in treatment options for patients suffering from edentulism and significant resorption of the alveolar ridge. These treatment options involve the utilization of custom subperiosteal implants. This paper looks at the historical evolution of these implants, the significance of diagnostic imaging, and the application of the contemporary methods of production, such as CAD-CAM and additive manufacturing. The research emphasizes the importance of accuracy and personalization provided by these emerging technologies that have rendered subperiosteal implants a more feasible and less intrusive alternative for patients suffering from significant bone loss.

## 1. Introduction

Tooth restoration using dental implants is a widely and commonly practiced procedure today. Efforts have been made to improve their efficacy and overall success rate over time. Dental implants can be classified based on material composition, bone interaction, available treatment options, and positioning within surrounding tissues [1]. Concerning their placement, in mandibular or maxillary bone tissue, dental implants fall into three categories: endosteal implants, transosteal implants, and subperiosteal implants (SPI). The severe bone atrophy of the edentulous maxilla or mandibula due to tooth loss, injury, or gum disease poses a challenge to achieving successful dental treatment [2]. For individuals facing this issue, various solutions have been devised, with subperiosteal implants being one option.

These implants, first introduced in the 1940s [3], were once popular for treating edentulous maxillary and mandibular arches, especially in cases of severe bone atrophy. Subperiosteal implants offer a solution for patients with significant alveolar arch resorption because unlike endosseous implants, which are embedded deep within the bone, they provide a framework that rests on top of the maxilla or mandible, beneath the periosteum. However, their popularity decreased over time due to challenges such as impression procedures, high infection rates, and difficulties in implant positioning. This decline led to a shift toward endosteal implants, driven by Dr. Branemark’s pioneering work on osseointegration [4].

The recent renewed interest in subperiosteal implants is a result of significant advancements in manufacturing technologies, such as additive manufacturing (3D printing), as well as developments in diagnostic imaging, in particular computed tomography (CT) coupled with digital planning software. The utilization of CT imaging data, for evaluation of alveolar ridge geometry and the creation of 3D models of the maxilla and mandible, in conjunction with computer-aided design (CAD) software (Geomagic Freeform version 2015.0.18), has led to the development of accurate implant designs. Moreover, the advancements in manufacturing technologies combined with materials such as titanium or titanium alloys have highly improved the quality of those implants. This has resulted in improved precision, fit, and durability of subperiosteal implants, making them a reliable and efficient choice for patients with atrophic arches. These advancements have overcome challenges, leading to increased predictability and better clinical outcomes [4,5]. The aim of this review is to provide a comprehensive overview of the evolution and advancements in custom subperiosteal implants for the treatment of partial or complete edentulism in patients with severe alveolar ridge atrophy. The structure of this review is organized into several focus areas: the historical development of subperiosteal implants, the significance of diagnostic imaging techniques, advancements in production methods, such as CAD-CAM and additive manufacturing, as well as the clinical outcomes and challenges associated with these implants.

## 2. Conventional Subperiosteal Implants

The evolution of subperiosteal implants traces back to the mid-20th century and has undergone substantial transformations over time. Dr. Dahl pioneered these implants in Sweden in 1942 by placing the first ever subperiosteal implant. The technique was later introduced in the United States during the 1940s by Goldberg and Gershkoff [3,6]. Initially, these implants were rudimentary and often faced issues due to inadequate bone exposure together with suboptimal material usage. This period was marked by designs and material choices that laid the groundwork for advancements in implant technology. Subperiosteal implants were less prevalent in the maxilla due to lower success rates, as well as unique characteristics that favored more stable prostheses. The reason for the low success rate of implants in the maxilla is mainly attributed to the poorer quality of bone tissue, which is predominantly cancellous. On the other hand, subperiosteal implants have shown better results in the lower jaw, where basal bone is abundant [7]. In the past, early implants encountered issues such as encapsulation, micromotion, bone loss, and high failure rates, with survival rates dropping as low as 50–60% at 15 years [8]. Factors such as cobalt corrosion and challenges with accurate positioning have led to a decline in their usage over time [8]. Markiewicz et al. [9] documented a case in which a subperiosteal implant made of chromium-cobalt alloy led to the development of an orocutaneous fistula due to a chronic infection. Subperiosteal implants have also faced obstacles, including increased infection risks due to sterilization practices, penetration into nasal and sinus regions due to stress on weaker bones, imperfections from the lost wax casting method, and difficulties with load distribution stemming from challenges in obtaining accurate bone impressions [4,10]. Key information from selected articles investigating conventional subperiosteal implants is summarized in Table 1.

### 2.1. Imaging Techniques

In the mid-20th century, the design and placement of conventional subperiosteal implants were primarily guided by traditional radiographic techniques and direct bone impressions taken during surgical exposure. This period, spanning from the 1940s to the 1980s, was marked by several challenges due to the limitations of the imaging technologies available at the time.

Initially, subperiosteal implants relied on two-dimensional radiographs and intraoral X-rays to assess the bone structure and plan the implant placement. These methods, although standard at the time, provided limited information about the three-dimensional anatomy of the jawbone. As a result, the accuracy of implant positioning was often compromised, leading to suboptimal fit and stability [3,6].

To obtain more precise details, surgeons had to perform invasive procedures, where the gingiva was incised, and direct impressions of the bone were taken. This process involved creating a physical mold of the exposed bone using materials such as dental plaster or rubber-based impression compounds [11,12,13]. A significant advancement occurred in 1985 when Truitt et al. [14] introduced a technique for designing subperiosteal implants for the mandible using computerized tomography (CT) scans to create a bone model before surgery. This resulted in a process that only required surgery for inserting the implant, making the procedure less invasive, i.e., a single stage [8].

### 2.2. Implant Design

Early designs of the maxillary subperiosteal implant relied on the hard palate for structural support, utilizing crossover struts. However, it soon became apparent that palatal soft tissues were unsuitable for resting on anything other than the palatal bone [7], which then led to the rapid abandonment of this initial design. Following versions of the maxillary subperiosteal implant encountered problems due to expansion into the maxillary sinus, with implant struts eventually settling and perforating the porous alveolar bone located beneath or next to the sinuses [7]. Removing struts and understanding that dense, stable bone should support the implant represented a significant leap forward in its development. Areas of dense, stable bone in the maxilla include the anterior nasal spine, canine fossas, and the palatal surface of the alveolar ridge. Nevertheless, these anatomical locations do not provide distal support. Therefore, in 1970, Linkow [15] modified the design to include the pterygomaxillary suture. Expanding on this idea in 1985, Cranin et al. [16] introduced the maxillary pterygohamular subperiosteal implant by utilizing pterygoid plates as buttresses [7,15,16]. 

### 2.3. Traditional Implant Materials

The initial subperiosteal implants described in the literature were constructed from various biomaterials. The materials used for subperiosteal implants were chromium, cobalt, and molybdenum alloys, with Vitallium being a well-known example, along with tantalum [8,11,12]. The first subperiosteal implants by Goldberg and Gershkoff in 1948 and Weinberg in 1950 were made from Vitallium [8,11]. These alloys were chosen for their reactive nature, strength, hardness, corrosion resistance, insolubility in bodily fluids, and biocompatibility. However, concerns about the side effects of these metals, resulting from them releasing ions into tissues, sparked the search for alternative solutions [8]. The field of subperiosteal implant research has made significant progress in recent decades, particularly in addressing the interaction between implants and human tissues. The release of metal ions into body tissues from implants is a key issue that has led researchers to explore various alternatives. At first, a carbon coating on implants was proposed, based on the supposed high biocompatibility of carbon [17]. This approach aimed to minimize the formation of connective tissue capsule around the implant. However, its adoption was limited because of inconclusive evidence of its efficacy at the carbon–tissue interface and potential adverse histopathological effects, as documented in a study where two carbon-coated subperiosteal implants cases were reported [18]. Later, Kay et al. [19] proposed another solution in the field of subperiosteal implants—the implementation of hydroxyapatite (HAP) coatings. These coatings were applied to the struts of subperiosteal implants. Hydroxyapatite (HA)-coated subperiosteal implants show a more attenuated response of the surrounding soft tissues than uncoated implants. When HA-coated implants are exposed due to a minor dehiscence of the wound, the affected area usually heals uneventfully [19]. This healing includes the initial development of granulation tissue, followed by the appearance of normal mucosal tissue, without the persistent inflammation often seen with non-HA-coated implants [19]. In addition, HA-coated implants are associated with a faster healing process around the implant struts. The conducted study expected that these coatings would improve bone–implant integration using the biocompatibility of ceramics and the mechanical characteristics of metallic components. HAP is a ceramic material that is known for its composition that closely resembles bone tissue and for its bioactive and osteoconductive properties [19]. Several studies have shown results conducted on HA-coated subperiosteal implants. For instance, research on 241 HA-coated mandibular subperiosteal implants revealed a survival rate of 98% over 7 years [20,21].

### 2.4. Manufacturing Technique

Early manufacturing techniques for subperiosteal implants primarily utilized the lost wax casting method. This process began with the creation of a wax model of the custom implant, which was meticulously crafted by hand. The wax model was then encased in a refractory material, forming a mold. The entire mold was subsequently heated to melt and remove the wax, leaving a cavity in the shape of the implant. Molten metal was then poured into this cavity to form the final implant. After the metal cooled, the refractory mold was removed, revealing a custom metal implant.

### 2.5. Surgical Procedures 

Dahl’s [3] as well as Goldberg and Gershkoff’s [6] initial implants were based on direct soft-tissue impressions, i.e., molds of the gingival tissues, which were later modified to match bone geometry on the basis of on intraoral X-ray image analysis. The height/thickness of gingival soft tissues was measured on intraoral radiographs and the bone model was modified accordingly [7,22]. In the 1950s, Berman et al. [23] introduced a two-stage surgical technique for implant procedures. The initial surgery involved incising and retracting the gingival tissues, followed by taking a direct impression of the bone using a custom tray that was made from earlier soft tissue impressions [7]. During this process, the tray was filled with a flexible material, such as rubber glue. Approximately 10 min later, the impressions were removed, and occlusal registration was performed. It is worth noting that in later studies, the use of prefabricated trays was omitted, opting instead to directly create bone impressions using materials such as polysulfides, silicones, and polyethers. In this procedure, the patient bites down on a baseplate and an occlusal rim is placed on the exposed mandible. The base of this occlusal rim is lined with soft wax to ensure that it adheres directly to the bone. This step is very important in determining the space between the upper and lower jaw and acts as a reference point for the height of the implant posts [8,24,25]. After taking the impression, a bone model is constructed using dental stone. This model is then set up with the correct vertical dimensions for creating an implant. Notably, this technique is known to cause considerable postoperative discomfort for patients, primarily due to the extensive exposure of the bone during the procedure [8,26].

### 2.6. Clinical Outcomes at Follow-Up

The findings of subperiosteal implants (SPIs) revealed technical shortcomings in early implants, as seen in Bodine’s assessment of 27 mandibular subperiosteal implants. Success rates dropped from 96% at 5 years, to a much lower 52% at 16 years [22]. Bloomquist et al. [26] highlighted that most implant failures were attributed to technical challenges. Their research involved 23 implants that were combined with bone grafts, and they emphasized issues related to the mucosal–metal junction as a critical problem area, leading to infections and implant failures. The method used was similar to conventional subperiosteal implants but with slight adjustments to the impression and surgical approach, showing a success rate of 68% after five years. Despite facing challenges, certain studies indicated success, with some patients retaining their implants for up to 21 years [22]. Nevertheless, challenges such as inadequate or non-rigid fixation, difficulties in accurately positioning the implants, and high complication rates contributed to a decrease in the adoption of this form of dental restoration. Reports involving 15-year follow-up periods showed survival rates ranging from 50% to 60% [8,27]. A research study carried out at the University of Southern California offered a perspective on subperiosteal implants and their longevity over a period of 21 years. It presented a survival rate of 79% at ten years and 60% at fifteen years, indicating that while effective in the short term, these implants exhibited reduced long-term viability over time. These results highlight the importance of assessment and adjustment of technology and techniques to enhance long-term results [28].

**Table 1:** Summary of selected articles (conventional subperiosteal implants).

Authors/Year of Publication	Title	No. of Implants	Implant Material	Imaging	Design and Manufacturing	Surgical Technique	Follow-Up
Weinberg, 1950 [11]	Subperiosteal implantation of a Vitallium (cobalt-chromium alloy) artificial abutment	2	Vitallium (a cobalt-chromium alloy), with a focus on utilizing a mesh-like structure for implantation.	-	The design involved a mesh-like structure made of Vitallium, intended to allow periosteal fiber growth through it, providing strength and stability. Manufactured using a casting method.	Two-staged surgical technique.	The follow-up included radiographs and clinical evaluations. One implant remained intact after almost one year of follow-up, the other had to be removed and inserted again due to complications.
Obwegeser, 1959 [12]	Experiences with subperiosteal implants	35	Chromium-cobalt-molybdenum alloys, especially Vitallium and tantalum.	-	Lost wax casting method.	Two-staged surgical technique.	The follow-up included radiographs and clinical evaluation. After one to three years, 2/3 of the patients experienced complications. Some of the implants had to be removed.
Kratochvil and Boyne, 1972 [13]	Combined use of subperiosteal implant and bone marrow graft in deficient edentulous mandibles: A preliminary report	1	Chrome-cobalt alloy.	-	The implant was designed as a mandibular chrome-cobalt casting, providing space between the existing bone and the implant structure for the bone marrow graft.	Two-staged surgical technique (exposure of the bone/impression taking, followed by insertion of the implant with the bone graft packed around it).	Initial clinical trial of this technique was observed for 14 months and has been described as most encouraging.
Bodine, 1974 [22]	Evaluation of 27 mandibular subperiosteal implant dentures after 15 to 22 years	27	Chrome-cobalt alloy.	-	Lost wax casting method.	Two-staged surgical technique.	Does not provide explicit details on how follow-ups were conducted for the subperiosteal implants. Study provides statistical analysis with success rates of subperiosteal implants: 96% at 5 years to 52% at 16 years.
Bloomquist, 1982 [26]	Long-term results of subperiosteal implants combined with cancellous bone grafts.	19	Not specified.	-	The implant contained removable abutments. Modifications in impression technique to decrease bone exposure.	Two-staged surgical technique. Minor modifications were made to the original technique to improve outcomes.	Evaluation conducted radiographically and clinically. Overall, 5-year success rate—68% (13 out of 19 implants).
Hess, 1982 [18]	Two cases of incompatibility to carbon-coated subperiosteal implants	2	Vitallium coated with vapor-deposited isotropic carbon.	-	Carbon-coated Vitallium: thickness of coating approximately 1 μm.	Two-staged surgical technique.	Follow-up evaluation involved monitoring of the patient’s response to the subperiosteal implant after its placement. Initial healing without complications; later episodes of swelling and pain, partial removal of mandibular implant.
Key, 1987 [19]	Hydroxyapatite-coated subperiosteal dental implants: Design rationale and clinical experience	339	Vitallium coated with hydroxyapatite (HA-coated).	CT scan (82 implants)	HA-coating applied to metal struts of the implant.	339 units placed, 257 were two-stage and 82 single-stage CT-scan procedures.	Single-stage surgery with CT scans appeared to provide the benefits of less invasiveness and less trauma to the patient compared to conventional two-stage surgery without CT scans.
Truitt, 1988 [29]	Use of computer tomography in subperiosteal implant therapy	41	Material not explicitly stated.	CT	Utilizes data from CT scans for accurate design and manufacturing.	This article delineates the transition from the traditional method of direct bone impression to the use of CT scan in order to create more accurate and reliable subperiosteal implants.	Specific details about the follow-up protocol were not revealed. Nevertheless, over the span of 2 years, the method of using CT scans to obtain a cast for SPI therapy has proven to be extremely reliable.
Fischer, 1993 [30]	CAD/CAM subperiosteal implants in Australia: Case report	1	Material not explicitly stated.	CT	Utilizes data from CT scan and CAD/CAM technology used for design and manufacturing.	Use of CAD/CAM technology to eliminate the first stage of surgery.	Follow-up details not mentioned, article encourages further research related to this technique.
Moore and Hansen, 2004 [20]	A descriptive 18-year retrospective review of subperiosteal implants for patients with severely atrophied edentulous mandibles	40	Chrome-cobalt alloy (Vitallium).	Panoramic radiographs	Lost wax casting technique; later using CT and stereolithography.	38 patients recieved two-staged surgery treatment with bone exposure and impression. 2 patients underwent single-stage CT-scan procedures.	The review of radiographs did not show any evidence of bone resorption under an abutment or major strut. The patients were clinically monitored over a period ranging from 2 to 18 years (average 8 years), with 14 patients having the implants for over 10 years, 12 patients between 5 and 10 years, and 11 patients for less than 5 years.

## 3. Modern Subperiosteal Implants 

Modern subperiosteal implants represent a significant advancement over traditional methods, largely due to innovations in diagnostic imaging and manufacturing technologies. The integration of advanced imaging techniques, such as CT and CBCT, has enabled the development of highly accurate, patient-specific implants. These custom-made implants are designed using CAD software and manufactured using additive manufacturing techniques, such as direct metal laser sintering (DMLS). These methods allow for the production of complex, biocompatible titanium structures that offer improved fit, stability, and osseointegration. The use of these advanced materials and technologies has resulted in implants that are less invasive, have higher success rates, and offer greater patient comfort compared to their predecessors. Table 2 summarizes the key findings from studies on modern subperiosteal implants.

### 3.1. Advanced Imaging Techniques 

The use of imaging technologies, such as computed tomography (CT) and cone beam computed tomography (CBCT), has brought a significant change in the development of subperiosteal implants (SPIs). These advanced imaging modalities have greatly improved the planning process by providing a non-invasive method for acquiring detailed patient anatomy. As a result, it is now possible to treat patients using a single-stage process that does not require such traumatic procedures, such as direct bone impressions. This simplifies the process, making it more efficient and less invasive. The digital workflow usually starts with a detailed CT scan that provides DICOM data for diagnosis and treatment planning. CAD-CAM systems are used to analyze these data to generate a 3D resin model, which helps in reconstructing patients’ bone structure and designing an implant guide plate. Ultimately, this results in the production of custom-made implants, specifically tailored to meet each patient’s unique anatomical needs, improving the fit and efficacy of the implant [4,31]. CBCT scans offer benefits over traditional CT scans, such as reduced radiation exposure, shorter examination times, and minimized image distortion caused by patient movement. They provide detailed three-dimensional images of a patient’s anatomical structures, allowing for precise evaluation of bone structure and density [32,33]. Al Ekrish and Ekram’s [34] research revealed an error of approximately 0.49 mm in CBCT data, while Suomalainen et al. [35] demonstrated measurement inaccuracies ranging from 2.3% to 4.7%.

### 3.2. Implant Design

Over the past twenty years, advancements in diagnostic imaging technology, in particular computed tomography, have ushered in a new digital age for dentistry. This era is marked by progress in 3D visualization with the use of volumetric imaging in assessing maxilla-facial tissues, in particular, bone tissue These modern imaging methods provide much more detailed, multiplanar imaging data of patient anatomy, which can be used to create detailed virtual models of the facial skeleton. In turn, these 3D models can be suitably modified and later used in CAD software to plan complex surgeries and design patient-specific implants.

The process begins with acquiring detailed three-dimensional images of the patient’s jaw using CT or CBCT imaging techniques. These images are converted into Digital Imaging and Communications in Medicine (DICOM) files and imported into specialized CAD software, such as Mimics (Materialise) and 3Matic (Materialise), to reconstruct the bone anatomy in 3D. The use of such software allows for the manipulation of the 3D bone model to design the implant, ensuring it conforms precisely to the bone contours, selecting optimal locations for fixation screws, and designing the prosthetic abutments [36,37]. Once the design is finalized, the CAD model is exported as an STL (stereolithography) file, which is used by additive manufacturing machines to create the physical implant. Technologies such as direct metal laser sintering (DMLS) are commonly employed to fabricate the implant from biocompatible materials, such as titanium. The precision of CAD/CAM ensures that the final product matches the digital model exactly, reducing the margin for error and improving the fit and function of the implant [37]

In recent years, there have been numerous developments in additive manufacturing technologies, i.e., 3D printing, in particular, powder bed fusion technologies, such as selective laser sintering (SLS), direct metal laser sintering (DMLS), and electron beam melting. These methods use either a laser or electron beam to melt and fuse layers of material powder together. Using such technology, it is possible to create complex, custom-designed maxillo-facial prostheses from biocompatible metal alloys tailored to each patient’s unique anatomical requirements [27,38,39]. This digital transformation has revitalized practices such as subperiosteal implants by integrating them with contemporary digital approaches, as follows:

- First, assessment of patient anatomy using 3D imaging techniques and creating a detailed 3D model of the facial skeleton.
- Second, implant design using computer software based on the unique patient anatomy and their specific treatment requirements.
- Third, use of additive manufacturing technologies and biocompatible materials’ metal alloys to ensure safety and compatibility with human tissues [4].

**Figure 1:**
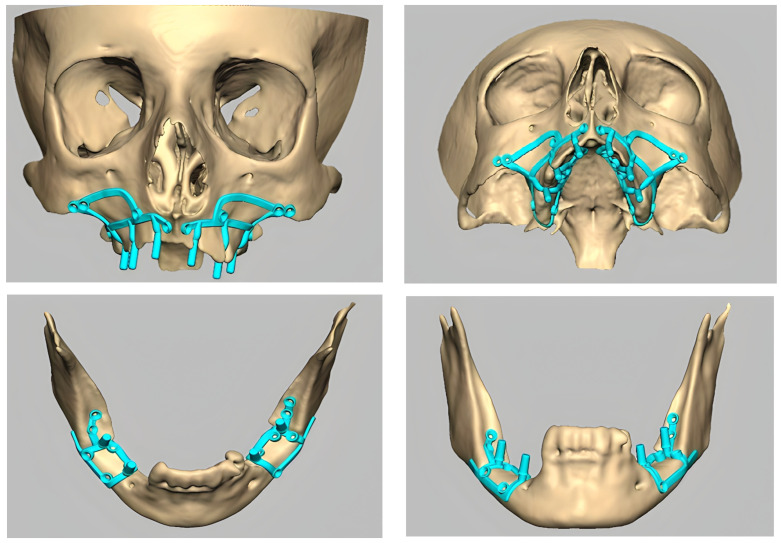
Examples of maxillary and mandibular subperiosteal implants.

These new-generation subperiosteal implants represent an advancement over their predecessors, as they consist of custom-made meshes or lattice-like structures that are precisely adapted to fit each patient’s bone geometry (Figure 1). This level of customization proves beneficial for treating edentulous alveolar arches by offering unprecedented precision and personalization compared to earlier implant technologies. As a result, these modern implants not only uphold the functionality of traditional implants but also enhance patient comfort and surgical outcomes [40]. 

### 3.3. Modern Implant Materials

Titanium: Titanium is highly valued for its biocompatibility, strength, and corrosion resistance. It forms a stable bond with bone, a process known as osseointegration, which is essential for the long-term stability of implants. Its properties facilitate a bond with the bone while reducing the footprint of the implant’s baseplate, resulting in reduced invasiveness, improved outcomes, and quicker recovery. Recent advancements have been made possible by using different materials and safer fabrication techniques. Titanium implants often develop a titanium oxide layer on their surface, which aids in healing by promoting protein adsorption, stabilizing blood clots, and ultimately integrating with bone tissue. These subperiosteal implants are primarily made from pure titanium or titanium alloys [4,31,36].

Polyether ether ketone (PEEK): Polyether ether ketone (PEEK) is a high-performance polymer known for its excellent mechanical properties and biocompatibility. It has a modulus of elasticity similar to that of bone, which helps in reducing stress shielding and promotes better load distribution. PEEK is also inert, reducing the risk of adverse reactions, and it does not interfere with imaging techniques, such as MRI or CT scans [41]. PEEK is used in cases where metal implants might not be suitable, such as in patients with metal allergies. It can be used alone or combined with other materials to optimize the mechanical stability and biocompatibility. Surface modifications, such as coating with bioactive materials or increasing surface roughness, have been developed to enhance its osseointegration capabilities. These modifications help PEEK implants achieve better integration with bone tissues, making them a viable alternative to titanium in certain clinical situations [37]. The integration of these advanced materials and design technologies has significantly improved the outcomes of subperiosteal implants. By utilizing the unique properties of titanium and PEEK, modern subperiosteal implants offer enhanced biocompatibility, reduced invasiveness, and improved long-term stability [36]. 

### 3.4. Computer-Aided Manufacturing Technologies

The introduction of additive manufacturing (AM) and 3D printing technologies, such as electron beam melting (EBM), selective laser melting (SLM), and selective laser sintering (SLS), has significantly revolutionized dental implant production. Advanced manufacturing techniques are used to create implants that precisely match their design specifications, ensuring they fit perfectly with a patient’s bone structure. This is essential for the stability and long-term survival of the implant. Additionally, the inherent flexibility of 3D printing in making intricate shapes allows for customizing implants to meet each patient’s needs, reducing the chances of implant failure [33]. The use of direct metal laser sintering (DMLS) technology has made it possible to produce pure titanium implants or titanium alloy implants, such as Ti-6Al-4V ELI, with features such as a porous surface that promotes osseointegration. In this process, bone cells grow onto the implant, creating a strong bond. In DMLS, layers of titanium powder are meticulously fused together using a laser beam. This process is repeated, layer-by-layer, allowing for the creation of highly detailed and complex implant structures that were previously unachievable. As a result, DMLS ensures implants with enhanced strength, durability, and integration capabilities [4,8,31]. Following the manufacturing process, each undergoes meticulous finishing touches, such as electro-erosion techniques, to optimize its fit and functionality. Gamma ray sterilization is the step to ensure the safety of the implant for surgical use. This technique represents a precise method of cleaning and sterilization when compared to earlier methods. It guarantees that the frameworks are thoroughly purified and biologically safe, thereby significantly reducing the likelihood of infection following implantation [4,31]. 

### 3.5. Surgical Procedures 

The surgical procedure for placing modern subperiosteal implants (SPIs) has undergone significant advancements, resulting in a more efficient, less invasive, and highly precise process compared to conventional methods. Below is a detailed description of the current surgical protocol and its benefits.

Surgical Procedure Steps:

1. Anesthesia: To ensure patient comfort throughout the procedure, local anesthesia is administered. Typically, 2% mepivacaine with 1:100,000 adrenaline is used for hemostasis. Each 1.8 mL cartridge contains 36 mg of mepivacaine hydrochloride and 18 mg of adrenaline, providing effective anesthesia and minimizing intraoperative bleeding [42].
2. Patient preparation: The patient is prepared following standard surgical protocols, including scrubbing and draping with povidone-iodine surgical scrub. This preparation maintains a sterile environment and minimizes the risk of infection [42].
3. Incision and flap design: A pyramidal flap is raised using three incision lines. The crestal incision is placed toward the palatal aspect of the crest of the ridge, made between the two teeth bounding the edentulous area. Two oblique releasing incisions are made at the distal ends of the crestal incision, allowing for adequate exposure of the bone [42].
4. Bone exposure and implant placement: After exposing the bone, the custom-made titanium subperiosteal implant is positioned on the bone surface. The implant is then secured using 2.0 mm grade-five titanium screws, ensuring a stable and precise fit, which enhances the implant’s integration with the bone [42].
5. Verification and adjustment: Following the placement of the implant, the surgeon verifies its fit and stability. Any necessary adjustments are made to ensure the implant is correctly positioned and will function effectively once the surgical site heals.
6. Suturing: The surgical site is closed using 3-0 Vicryl sutures. This promotes proper healing and protects the implant from exposure to the oral environment during the initial healing phase [42].

### 3.6. Clinical Outcomes at Follow-Up

In the research conducted by Dimitroulis et al. [31], postoperative monitoring was integral in ensuring the wellbeing of patients who received implants. Each patient underwent an evaluation using X-rays to detect any issues, such as broken screws or any compromise in the implant’s structural stability. Follow-up appointments involved the removal of prostheses to check for signs of infection discharge pockets around the posts or any indications of wound separation, which could expose the implant framework. Success in evaluating implants was based on factors such as the patient’s comfortability to chew without pain, normal speech patterns, and satisfactory appearance. Criteria related to implants included ensuring no exposure of the metal baseplate, absence of movement, no clinical infections, and no X-ray evidence suggesting loosened screws or fractured prostheses. The presence of pink, keratinized gingiva surrounding each transmucosal post was also noted as a key indicator of success. The effectiveness of subperiosteal implants relies heavily on their ability to adhere functionally to bone. The process of osseointegration, where bone cells connect to the surface, is quite intricate and influenced by numerous factors [43]. These factors include the material and surface properties of the implant, the congruence between the implant and bone, and the surgical techniques used. A lack of direct contact between the implant and bone often leads to fibrous integration instead of osseointegration [8]. Studies mentioned here have shown results in terms of outcomes. Some common issues reported include pain, swelling, and inflammation. The removal of a subperiosteal implant poses a highly complex prosthodontic challenge. Several studies have noted cases where implants needed to be removed during follow-up periods. A few studies indicated results where there were minimal complications over time and the implants remained stable. However, it is important to note that this does not automatically imply that the implants were entirely successful. Recent advancements in technology, such as direct metal laser sintering (DMLS) and CAD-CAM technologies, with 3D metal printing, have significantly improved their reliability and long-term efficacy. These implants have proven to be a treatment choice for patients with atrophic arches. A retrospective study involving 70 patients who received implants showed a survival rate of around 96% after a two-year follow-up period [8,27]. Another study on the generation of implants utilizing CAD-CAM technologies and 3D metal printing demonstrated promising outcomes. Over 4 years, 21 such devices were implanted, with a success rate of 85.7%. Many patients reported improvements in comfortability with chewing, speech, and overall quality of life [31]. Additionally, a study by Mangano et al. [37] in 2020 evaluated the performance of ten implants manufactured using DMLS technology. Despite challenges with two implants, all implants remained functional after one year, resulting in a 100% survival rate. These studies highlight the advancements in technology and showcase the improved effectiveness and patient satisfaction [8].

**Table 2:** Summary of selected articles (modern subperiosteal implants).

Authors/Year of Publication	Title	No. of Implants	Implant Material	Imaging	Design and Manufacturing	Surgical Technique	Follow-Up
Kusek, 2009 [44]	The use of laser technology (Er;Cr:YSGG) and stereolithography to aid in the placement of a subperiosteal implant: case study	1	Custom-fabricated titanium framework.	CT and 3D modeling	CAD/CAM and additive manufacturing technology for fabricating the titanium implant.	Single-staged surgical technique.	Article does not provide specific details about post-surgery follow-up information.
Mounir, 2017 [42]	Titanium and polyether ether ketone (PEEK) patient-specific subperiosteal implants: two novel approaches for rehabilitation of the severely atrophic anterior maxillary ridge	10	Titanium (Grade 23 Ti-6Al-4V ELI), PEEK.	CT, CBCT	CAD/CAM, electron beam melting (EBM).	Single-staged surgical technique.	Monthly follow-up for 12 months; postoperative instructions, including medication and oral hygiene.
Cerea and Dolcini, 2018 [45]	Custom-made direct metal laser sintering titanium subperiosteal implants: A retrospective clinical study on 70 patients	70	Direct metal laser sintering (DMLS) titanium.	Preoperative CBCT scan and digital planning	Custom-made using direct metal laser sintering (DMLS).	Single-staged surgical technique.	Two-year follow-up, 95.8% survival rate, and low complication rates.
Mangano, 2020 [37]	Custom-made 3D-printed subperiosteal titanium implants for the prosthetic restoration of the atrophic posterior mandible of elderly patients: a case series	10	3D-printed subperiosteal titanium.	Preoperative CBCT scan and digital planning	Custom-made using direct metal laser sintering (DMLS).	Single-staged surgical technique.	One-year follow-up, 100% survival rate, minor complications in 30% of patients (3 out of 10).
Nemtoi, 2022 [40]	Custom-made direct metal laser sintering titanium subperiosteal implants in oral and maxillofacial surgery for severe bone-deficient patients—A pilot study	16	Titanium (DMLS, Ti6Al4V).	Orthopantomography (OPT), CBCT	CAD/CAM, selective laser melting (SLM).	Single-staged surgical technique.	Monthly follow-up for six months; evaluation of fit, stability, and complications. The study reported a high implant survival rate of 93.75% over the six-month follow-up period.
Vatteroni, 2023 [4]	The new generation of subperiosteal implants for patient-specific treatment of atrophic dental arches: A literature review and two case reports	2	Direct metal laser sintering (DMLS) titanium.	Preoperative CBCT scan and digital planning	Custom-made using direct metal laser sintering (DMLS).	Single-staged surgical technique.	Panoramic radiograph 1 year after surgery shows good osseointegration.
Arshad, 2023 [33]	Additively custom-made 3D-printed subperiosteal implants for the rehabilitation of the severely atrophic maxilla (a case report)	1	Titanium alloy (Grade 23 Ti6Al4V-ELI).	CBCT	CAD/CAM, additive manufacturing (3D printing) using titanium alloy.	Single-staged surgical technique.	Follow-up for 3 years; minor dehiscence in two areas but no progression; no implant fractures.
Onică, 2024 [36]	Long-term clinical outcomes of 3D-printed subperiosteal titanium implants: A 6-year follow-up	61	Titanium alloy (DMLS, PowderRange Ti64).	CBCT (Green X, Vatech)	CAD/CAM, DMLS system.	Single-staged surgical technique.	Follow-up for 6 years; 9 of 36 cases were successful; 27 cases had complications, including early/delayed frame exposure, mobility, infections.

## 4. Conclusions

Subperiosteal implants have undergone advancements, from being a solution for atrophic posterior mandibles with challenges, such as requiring two separate surgeries and poor fit at the site, to a more patient-focused approach that utilizes modern technologies. The introduction of direct metal laser sintering (DMLS) has transformed this field significantly, particularly benefiting individuals with bone loss who are not suitable candidates for extensive bone regeneration procedures. DMLS technology ensures accuracy, resulting in implants that perfectly match patients’ anatomical needs. This helps minimize complications and improve success rates. While promising, these developments necessitate further comprehensive clinical studies for full verification of its widespread applicability. The modern approach involving DMLS simplifies the process significantly when compared to traditional methods. By eliminating the necessity for bone grafting, it not only reduces the surgical time but also simplifies operations. The careful design and creation of these custom implants tailored to each patient’s anatomy leads to improved outcomes and decreases the postoperative risks. As this technology advances, it has the potential to become a standard in handling cases among older individuals facing specific dental issues.
